# Sleeping, Waking, … and Glucose Homeostasis

**DOI:** 10.1371/journal.pbio.0020415

**Published:** 2004-11-02

We often think of ourselves as either a day person or a night person—one who rises with the sun, raring to go, or one who prefers to stay up through the night to get things done. Regardless, we each have our regular waking and sleeping cycles. It's been known for some time that variations in sleep and wakefulness are part of our circadian rhythm, or molecular clock. A portion of the brain called the hypothalamic suprachiasmatic nucleus (SCN) regulates this biorhythm. When this area of the hypothalamus is destroyed in animal models, the circadian rhythm is disrupted. Two transcription factors (proteins that regulate gene expression) called Bmal1 and Clock regulate aspects of circadian rhythm, possibly by regulating neurons in the SCN.

Other aspects of human physiology are also regulated in a circadian manner. Besides altering sleep and wakefulness patterns, ablation of the SCN alters the ability to regulate sugar levels. Sugar (glucose) levels must be maintained within fairly narrow limits for survival. This regulation is controlled in part by a balance between blood sugar level and insulin production (insulin lowers the blood sugar level). In people and in mouse models, both glucose level and insulin level are subject to circadian rhythms. It isn't clear, however, if this is a behavioral effect, whereby the disruption of the SCN might alter our feeling of being well fed—that is, being sated—as eating has a profound effect on blood sugar levels.

**Figure:**
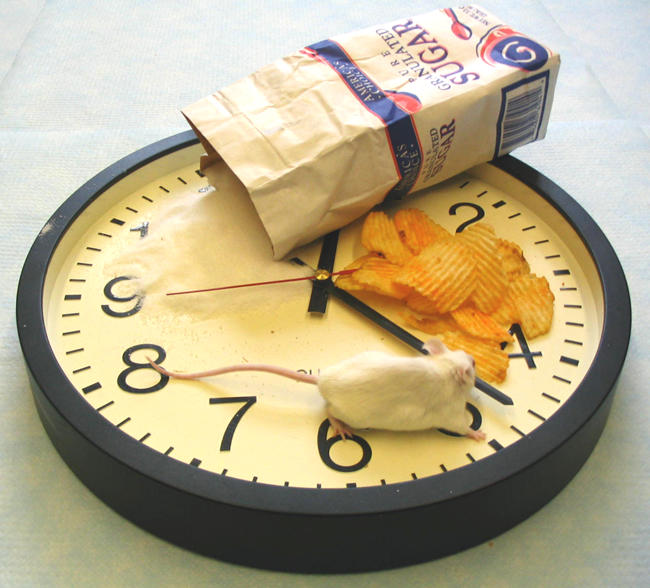
Metabolic clock regulation of glucose homeostasis

Garret FitzGerald and colleagues tested the effect of the molecular clock genes in glucose regulation (homeostasis) by examining mice in which Clock and Bmal1 were impaired. In normal mice they observed a peak in glucose levels early in the day. This diurnal regulation was lost in the mutant mice. Furthermore, whereas the normal mice could fairly easily return their glucose levels to normal when they were artificially treated with insulin, this ability was severely impaired in the mutant mice. What's more, a high-fat diet amplified this circadian variation in the normal animals, but the rhythm was abolished in the mutants on a high-fat diet. Thus, the authors demonstrated that circadian control of blood glucose levels is due directly to the presence of these transcriptional factors rather than due to some other behavioral effect that ablation of the hypothalamus might have caused. It's possible, therefore, that besides what we eat, our internal circadian clock could also be an important regulator of blood sugar levels.

What is still left to be explored is whether the change in glucose that results from disruption of the Clock and Bmal1 genes is due to the transcription factors' effect as circadian regulators or to an activity of these transcription factors that is unrelated to circadian rhythm generation. But the study does raise the possibility that when you eat may be as important to your health as what you are eating.

